# Calcitriol Modulates the CD46 Pathway in T Cells

**DOI:** 10.1371/journal.pone.0048486

**Published:** 2012-10-29

**Authors:** Karoline Kickler, Siobhan Ni Choileain, Anna Williams, Anna Richards, Anne L. Astier

**Affiliations:** 1 MRC Centre for Inflammation Research, University of Edinburgh, Queen's Medical Research Institute, Edinburgh, United Kingdom; 2 Multiple Sclerosis Research Centre, MRC Centre for Regenerative Medicine, The University of Edinburgh, Edinburgh Bioquarter, Edinburgh, United Kingdom; Institute Biomedical Research August Pi Sunyer (IDIBAPS) – Hospital Clinic of Barcelona, Spain

## Abstract

The complement regulator CD46 is a costimulatory molecule for human T cells that induces a regulatory Tr1 phenotype, characterized by large amounts of IL-10 secretion. Secretion of IL-10 upon CD46 costimulation is largely impaired in T cells from patients with multiple sclerosis (MS). Vitamin D can exert a direct effect on T cells, and may be beneficial in several pathologies, including MS. In this pilot study, we examined whether active vitamin D (1,25(OH)_2_D_3_ or calcitriol) could modulate the CD46 pathway and restore IL-10 production by CD46-costimulated CD4+ T cells from patients with MS. In healthy T cells, calcitriol profoundly affects the phenotype of CD46-costimulated CD4+ T cells, by increasing the expression of CD28, CD25, CTLA-4 and Foxp3 while it concomitantly decreased CD46 expression. Similar trends were observed in MS CD4+ T cells except for CD25 for which a striking opposite effect was observed: while CD25 was normally induced on MS T cells by CD46 costimulation, addition of calcitriol consistently inhibited its induction. Despite the aberrant effect on CD25 expression, calcitriol increased the IL-10:IFNγ ratio, characteristic of the CD46-induced Tr1 phenotype, in both T cells from healthy donors and patients with MS. Hence, we show that calcitriol affects the CD46 pathway, and that it promotes anti-inflammatory responses mediated by CD46. Moreover, it might be beneficial for T cell responses in MS.

## Introduction

CD46 is a regulator of complement activity that binds to the C3b and C4b complement components, allowing their cleavage by factor I [1]. CD46 also binds to several pathogens [2] and promotes autophagy upon pathogen binding providing a crucial step in the control of infections [3]. Moreover, CD46 is key in the regulation of the adaptive immune response. Costimulation with CD3/CD46 leads to increased T cell proliferation [4], regulates T cell mediated inflammation in a CD46-transgenic mouse model [5], induces morphological changes [6] and affects T cell polarity [7]. The enzymatic processing of CD46 is involved in the control of T cell homeostasis, by regulating not only activation but also termination of T cell responses [8], [9]. Importantly, CD46 costimulation promotes Tr1-like Treg differentiation, characterized by secretion of large amounts of IL-10 and low levels of IFNγ [10], [11]. Defects in IL-10 production upon CD46 activation have been demonstrated in patients with MS [12], [13], [14], asthma [15] and rheumatoid arthritis [11].

Vitamin D deficiency has been associated with a higher rate of several diseases including MS and asthma [16], [17], [18]. Active Vitamin D (1,25(OH)_2_D_3_ or calcitriol) has some immunoregulatory capacity, with reports of a direct action on T cells [19]. T cell activation induces the Vitamin D receptor (VDR) [20], [21], that is required for TCR signaling and T cell activation [22]. Calcitriol can decrease secretion of IFNγ [23], [24], [25], modulates IL-10 production and generates Tregs [26], [27], [28], [29], [30], [31], which are essential for immune homeostasis. Treatment with calcitriol suppresses the development and progression of EAE, the murine model of MS [29], [32], [33], and ameliorates several other models of autoimmune diseases [34]. In MS, Vitamin D supplementation is safe and has been associated with a modulation of T cell responses [35], [36], [37]. Although the role of Vitamin D on the immune system is intensively studied, no study, as far as we are aware, has investigated the role of calcitriol on CD46 functions. As CD46 costimulation is key in controlling IL-10 production and this pathway is defective in pathologies modulated by Vitamin D supplementation, we investigated whether calcitriol could modulate CD46 expression and function of activated T cells. The results of our pilot study show that calcitriol affects CD46 expression and strongly modulates T cell responses and the phenotype of CD46-activated T cells from both healthy donors and patients with MS. However, a striking difference was that CD46-costimulated MS T cells, in the presence of calcitriol, expressed much lower levels of CD25 compared to T cells isolated from healthy donors. Although MS T cells produce less IL-10 than cells from healthy donors, addition of calcitriol could restore a normal IL-10:IFNγ ratio in patients with MS. These data provide novel mechanisms of action of calcitriol that warrant further investigation of this pathway in the pathologies in which CD46 is dysfunctional.

## Methods

### Ethics Statement

Ethical approval was obtained from the Lothian Health Board Ethics Committee.

### Antibodies and reagents used

The following antibodies were used to activate T cells: anti-CD3 (OKT3, 5 µg/ml), anti-CD28 (CD28.2, 5 µg/ml), anti-CD46 (MCI.20.6, 10 µg/ml). Calcitriol was purchased from Sigma-Aldrich and used at 10^−7^M. Recombinant human IL-2 (Tecin) was added at 10 U/ml. The antibodies for flow cytometry were as follows: anti-CD46-FITC (clone MEM-258), anti-CD28-PE (clone 28.2), anti-OX40-FITC (clone Ber-ACT35), anti-PD-1-PE (clone EH12.2H7), anti-4-1BB/CD137-APC (clone 4B4-1) were purchased from Biolegend; anti-Foxp3-APC (clone PCH101; ebioscience); anti-CD25-APC (clone M-A251), anti-CTLA-4-PE (clone BNI3) were purchased from BD Pharmingen. The blocking anti-IL-10Rα was purchased from R&D.

### Cell purification and activation

PBMC were isolated from healthy donors (n = 15) or patients with MS in the relapsing-remitting stage (n = 11) (see Table 1). CD4+ T cells were negatively isolated (Miltenyi Biotec, purification >90%). In some instances, naïve T cells (Miltenyi) and CD8+ T cells were also purified (EasySep, StemCell, purification >96%) and studied. T cells (1×10^6^ cells/1ml/well) were then activated by culturing in 24-culture wells pre-coated with anti-CD3, anti-CD28, or anti-CD46, in RPMI containing 10% FCS [12]. Exogenous IL-2 (10 U/ml) was added to CD3/CD46 costimulated CD4+ T cells as previously described [10]. The active metabolite of vitamin D_3_ (1,25-Dihydroxyvitamin D3 (1,25(OH)_2_D_3_) or calcitriol) (10^−7^M) or similar dilutions of 95% ethanol as vehicle control was added to the culture.

**Table 1:** Characteristics of the donors used in this study as to sex, age and disease status.

	Healthy controls	Patients with MS
Sex	3M/12F	2M/9F
Age (yr, mean±SD)	33.2±6.02	41±8.37
Age range	23–48	27–51
Disease duration (yr, mean±SD)	-	10±9.3
Disease duration range (yr)	-	2–30
EDSS (mean±SD)	-	2.61±1.94
EDSS median	-	2
EDSS range	-	0–5.5
Treatment	-	7 IFNβ/ 3 untreated

### Proliferation assay

Purified T cells (5×10^5^ cells/200 µl/well) were cultured in 96-culture wells pre-coated with anti-CD3 or anti-CD46, in 10% FCS-RPMI 1640 for 72 hrs, before addition of 0.5 µCi of [^3^H]thymidine (Amersham). Proliferation was determined using a Liquid Scintillation Counter (Wallac).

### Suppression assays

Bystander purified T cells were pre-labeled with carboxyfluorescein diacetate succinimidyl ester (CFSE) (1 µM; Molecular Probes Invitrogen) according to manufacturer's instructions. Briefly, after 3 washes in cold PBS, T cells (1×10^6^/500 µl PBS) were stained with CFSE for 10 min at 37C, followed by 3 washes in cold 10% FCS-RPMI 1640 before being added to the wells and activated by anti-CD3 antibodies in presence of culture medium or of supernatants of activated T cells. In some experiments, addition of a blocking anti-IL-10Rαantibodies (10 µg) or control IgG1 was added. After 4 days, cells were harvested and cell division estimated by flow cytometry.

### Flow cytometry

The expression level of CD46, CD28, CD25, Foxp3 and CTLA-4 on CD4+ and naïve CD4+ T cells was assessed by flow cytometry. The levels of OX40, 4-1BB/CD137 and PD-1 were also determined on CD8+ T cells. The relative expression was calculated by calculating the ΔMFI (geometric MFI antibody stained – geometric MFI control antibody), except for Foxp3 for which the % of Foxp3-expressing cells is represented. Data were acquired on a FACSCalibur flow cytometer and analyzed with FlowJo™ software.

### Cytokine production

Cytokine production was determined in cell culture supernatants using ELISA specific for human IL-10 (BD Pharmingen) and IFNγ (Endogen, Thermofisher), as previously reported [12]. Recombinant hIL-10 (BD Pharmingen) and hIFNγ (Endogen) were used as standards. Alternatively, cytokine secretion was also determined using the IL-10 and IFNγ secretion assays from Miltenyi using anti-IFNγ-APC and anti-IL-10-PE detection reagents, according to the manufacturer's instructions.

### Statistical analyses

The groups using different donors were analyzed using Graphpad Prism software: data were analyzed using the Wilcoxon test, a non-parametric test, when comparing paired samples from the same donors; data were analyzed using the Mann Whitney U-test when comparing data obtained from patients and healthy donors. Elisa data obtained from the same experiment were analyzed using Excel Student's t-test. All p-values are two-tails and with a 95% confidence interval. Mean and SEM are represented.

## Results

### Active Vitamin D (1,25(OH)_2_D_3_ or calcitriol) modulates CD46 expression

**Figure 1:**
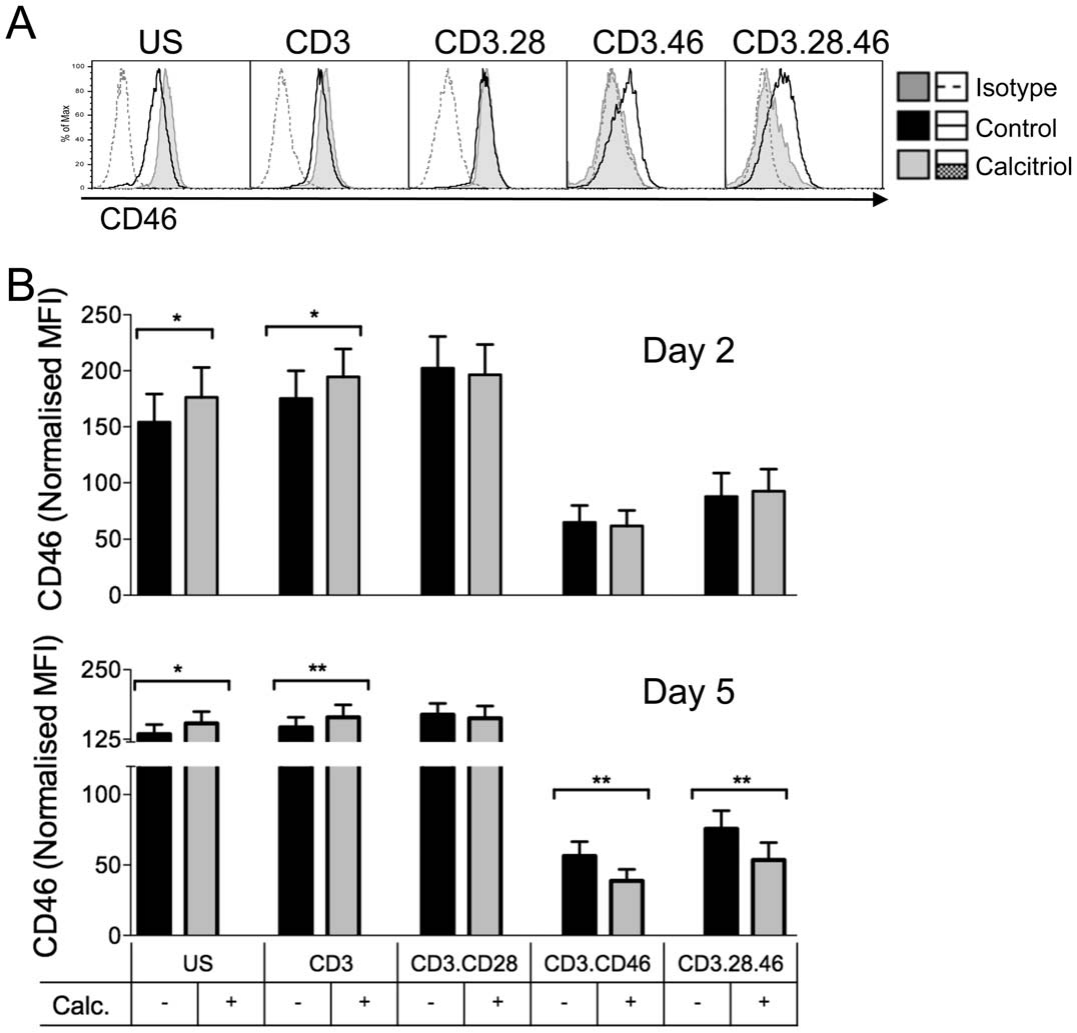
Calcitriol modulates CD46 expression in CD4+ T cells. Purified CD4+ T cells from healthy donors were left unstimulated (US), or were stimulated by immobilized anti-CD3, anti-CD3/CD28, or anti-CD3/CD46 antibodies as indicated in presence of calcitriol (10^−7^M) or ethanol as vehicle control. CD46 expression was monitored by flow cytometry. The representative expression of CD46 after 5 days of culture is shown in (**A**). The average expression of CD46 detected after 2 or 5 days for the different donors analyzed (mean ± SEM; n = 15) is shown in (**B**). Samples were analyzed using the Wilcoxon test.

We have recently shown that expression of CD46 at the cell surface of CD4+ T cells is tightly regulated by T cell activation, and that cleavage of CD46 ectodomain occurred upon its ligation in activated T cells [8], [9]. Hence, we first determined whether addition of active Vitamin D could affect the expression of cell surface CD46. CD4+ T cells were purified from blood of healthy donors and activated with immobilized anti-CD3, anti-CD3/CD46 or anti-CD3/CD28 antibodies in presence of calcitriol (10^−7^M, as previously described in [38]) or ethanol as a vehicle control. Figure 1A shows representative data from one donor after 5 days of culture, and the average results obtained for the different donors after 2 or 5 days of culture are shown in Figure 1B (n = 15). As previously reported [9], CD46 expression is strongly downregulated upon its own triggering. Addition of calcitriol further decreased CD46 surface expression (Figure 1A). Calcitriol had not much effect at day 2, although it slightly increased the levels of CD46 in unstimulated and CD3-activated T cells (Figure 1B). In contrast, at day 5, calcitriol significantly decreased CD46 expression upon CD46 costimulation. This effect was CD46 dependent, as no significant effect of calcitriol on CD46 expression was noticed when cells were costimulated by CD28 (Figure 1).

### Calcitriol modulates the phenotype of CD46-activated CD4+ T cells but differently so in MS T cells

**Figure 2:**
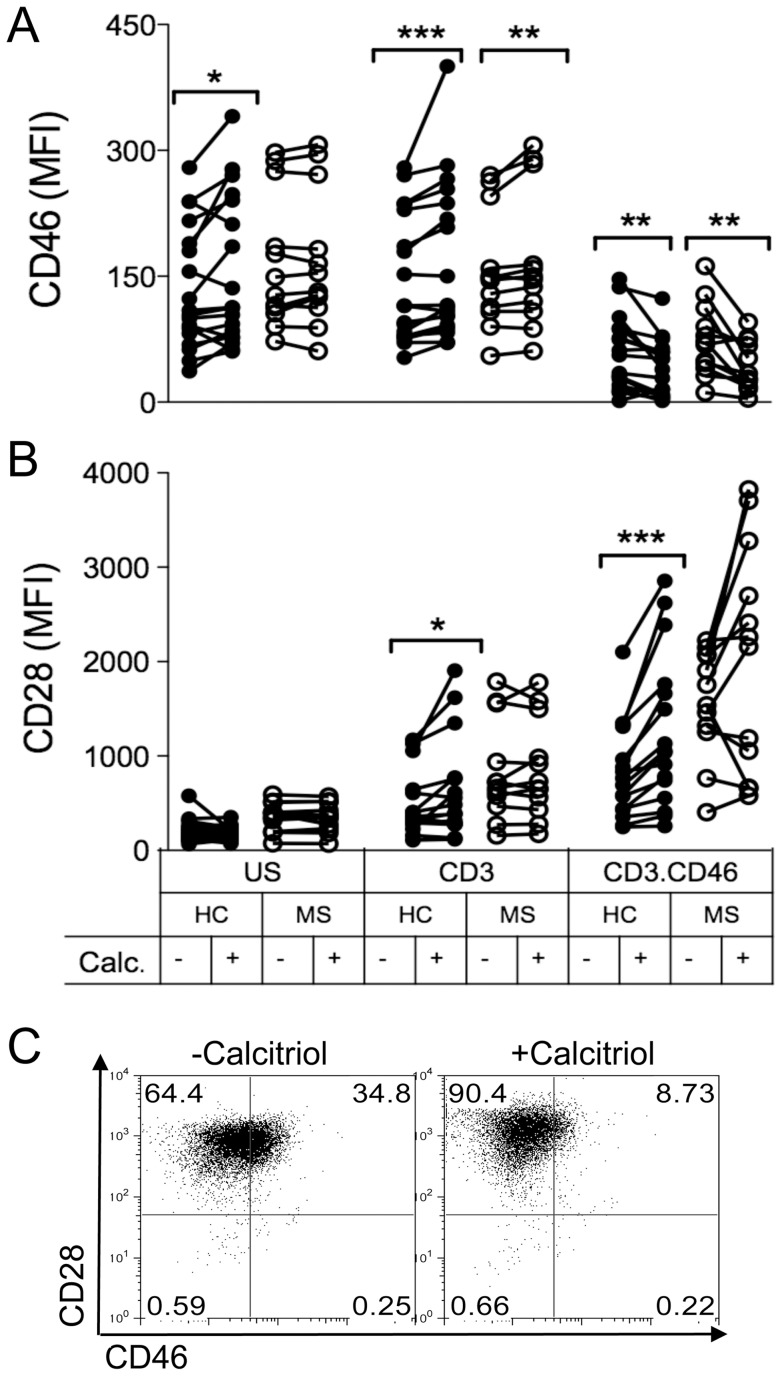
Calcitriol promotes concomitant CD46 downregulation and CD28 upregulation in CD46-costimulated CD4+ T cells. Purified CD4+ T cells from a cohort of RRMS patients (MS) (n = 11) or healthy controls (HC) (n = 15) were stimulated by immobilized anti-CD3 or anti-CD3/CD46 antibodies as indicated in presence of calcitriol (10^−7^M) or ethanol as vehicle control for 5 days. Expression of CD46 (**A**) or CD28 (**B**) was monitored by flow cytometry. Samples were analyzed using the Wilcoxon test. (**C**) The dot-plot showing the concomitant changes in expression of CD46 and CD28 following calcitriol treatment on CD46-costimulated T cells is represented for one healthy donor.

We next compared the effects of calcitriol on the phenotype of activated CD4+ T cells isolated from the blood of a cohort of healthy donors (n = 15) or of patients with MS in the relapsing-remitting stage (RRMS, n = 11). Purified CD4+ T cells were activated with anti-CD3 or anti-CD3/CD46 antibodies for 5 days in presence of calcitriol or ethanol, and expression of CD46 (Figure 2A) and of CD28 (Figure 2B), one of the main costimulatory molecules for T cells, was monitored. Similar effects of calcitriol on CD46 expression were observed in both cohorts, with a decrease in CD46 expression observed upon its ligation. Strikingly, the decrease in CD46 expression observed upon CD46 costimulation was correlated with a strong increase in CD28 levels (Figure 2C). An increased CD28 expression could also be detected after 2 days (not shown). A similar increase in CD28 expression levels was detected in most MS T cells (7 out of 11), but 4 patients exhibited a lower CD28 expression upon CD46 costimulation in presence of calcitriol (Figure 2B). Moreover, a slight increase in CD28 was also detected with calcitriol upon CD3 activation alone for most healthy T cells but not for MS T cells, suggesting an abnormal regulation of CD28 expression by calcitriol in some MS T cells.

**Figure 3:**
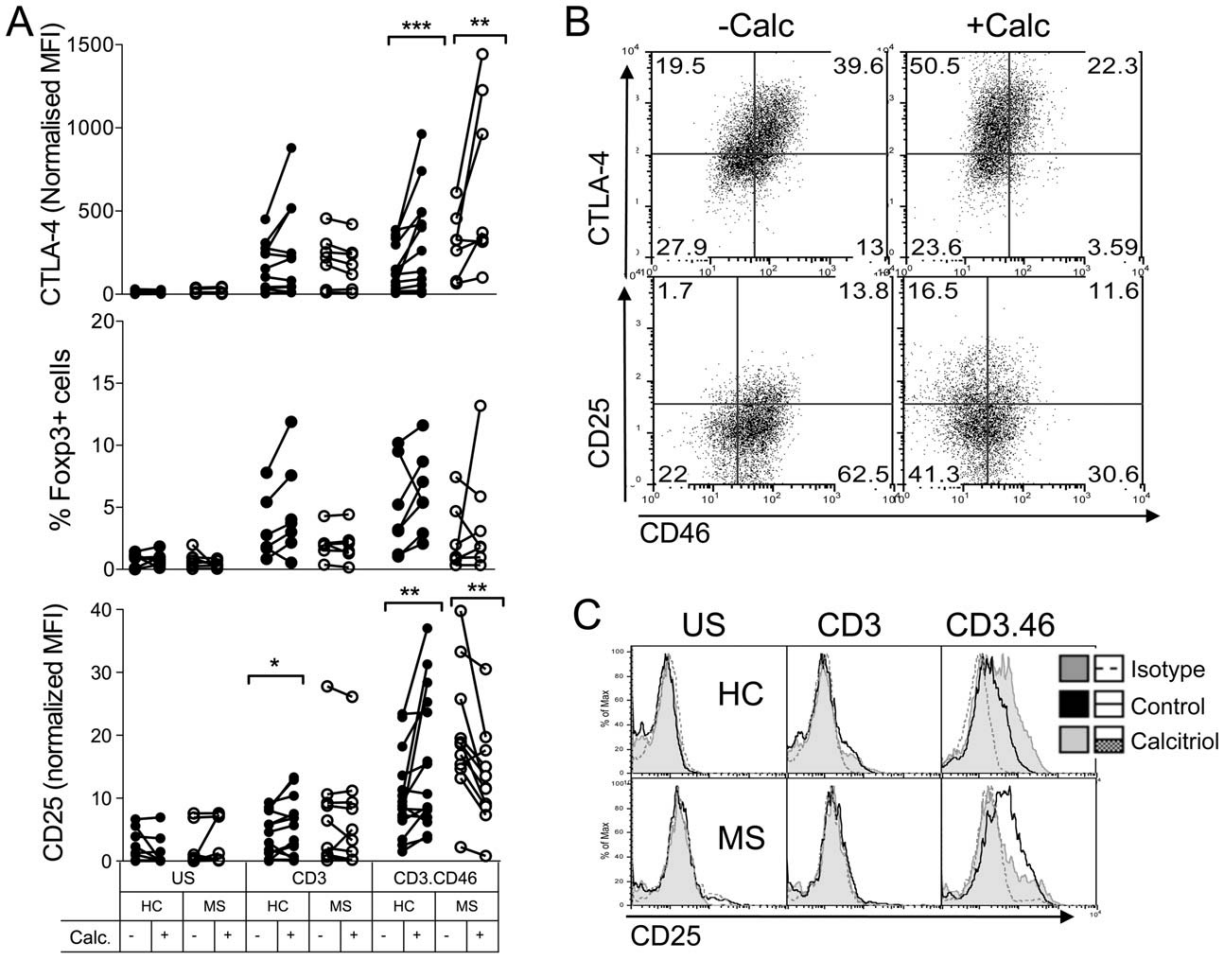
Calcitriol promotes CTLA-4, Foxp3 and CD25 expression on CD4+ T cells but not in MS. (**A**) Purified CD4+ T cells from MS patients or healthy controls (HC) were left unstimulated or stimulated by immobilized anti-CD3 or anti-CD3/CD46 antibodies in presence of calcitriol (10^−7^M) or ethanol, for 5 days. Expression of CTLA-4, Foxp3 and CD25 was then monitored by flow cytometry. (**B**) The representative data showing the concomitant increase in CD25 or CTLA-4 with the downregulation of CD46 upon CD3/CD46 costimulation for one healthy donor are shown. (**C**) The representative data showing changes in CD25 expression following addition of calcitriol in one healthy donor and one patient with MS are shown.

We also determined the expression levels of CTLA-4, Foxp3 and CD25, known to be upregulated by CD46 costimulation [9] and calcitriol [28]. In CD4+ T cells isolated from healthy donors, addition of calcitriol promoted CTLA-4, Foxp3 and CD25 expression in CD46-activated T cells at day 5 (Figure 3A). The increase in CTLA-4 and CD25 was correlated to the decrease in CD46 expression (Figure 3B). A similar increase in CTLA-4 was detected in MS T cells. Foxp3 expression was only induced on some donors and lower levels were observed than in healthy T cells. The most striking effect was observed for CD25. While CD3/CD46 costimulation was able to induce CD25 expression, the addition of calcitriol significantly decreased CD25 expression in all MS T cells except for one donor (Figure 3A). This defect seems to be specific of CD46 costimulation as not observed upon CD28 costimulation (Fig. S1). Figure 3C shows representative data obtained for one healthy control and one patient with MS. Hence, calcitriol exerts a profound effect on CD46-costimulated CD4+T cells, by modulating CD46, CD28, CD25, CTLA-4 and Foxp3 expression, but the modulation of expression of CD25, and in some patients of CD28, is aberrant in MS T cells upon CD46-costimulation.

### Calcitriol promotes the switch from IFNγ-producing to IL-10-secreting cells

**Figure 4:**
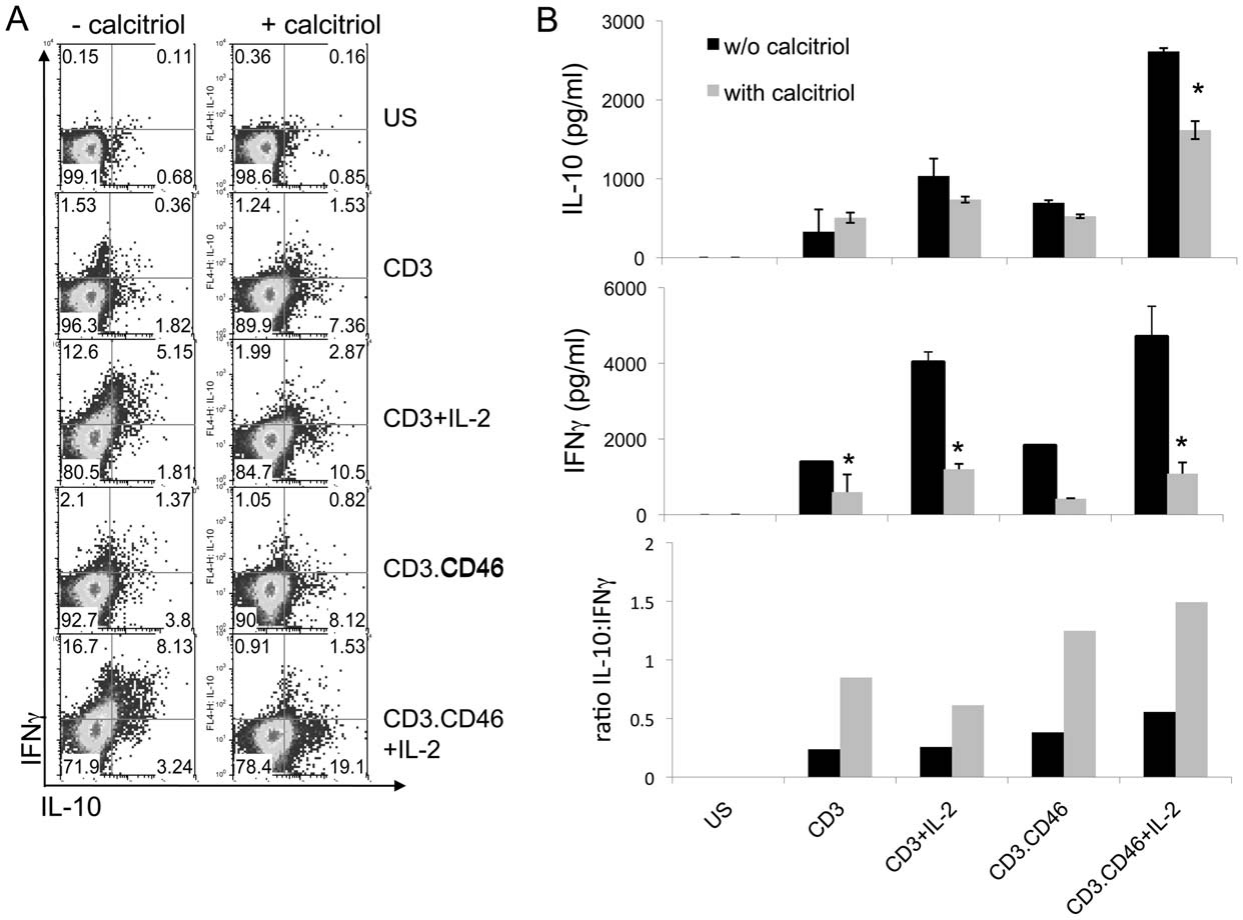
Calcitriol allows the switch from Th1 to Tr1 in CD4+ T cells. (**A**) Purified CD4+ T cells from one healthy donor were left unstimulated or stimulated as indicated by immobilized anti-CD3 and anti-CD3/CD46 antibodies, in presence or absence of IL-2 and calcitriol. After 2 days, the production of IL-10 and IFNγ was assessed using the secretion catch assay (Miltenyi). (**B**) The production of IL-10 and IFNγ in the supernatants from the same wells was also determined by ELISA. The IL-10:IFNγ ratio is also represented (mean ± SEM; samples were analyzed using Student's t-test).

**Figure 5:**
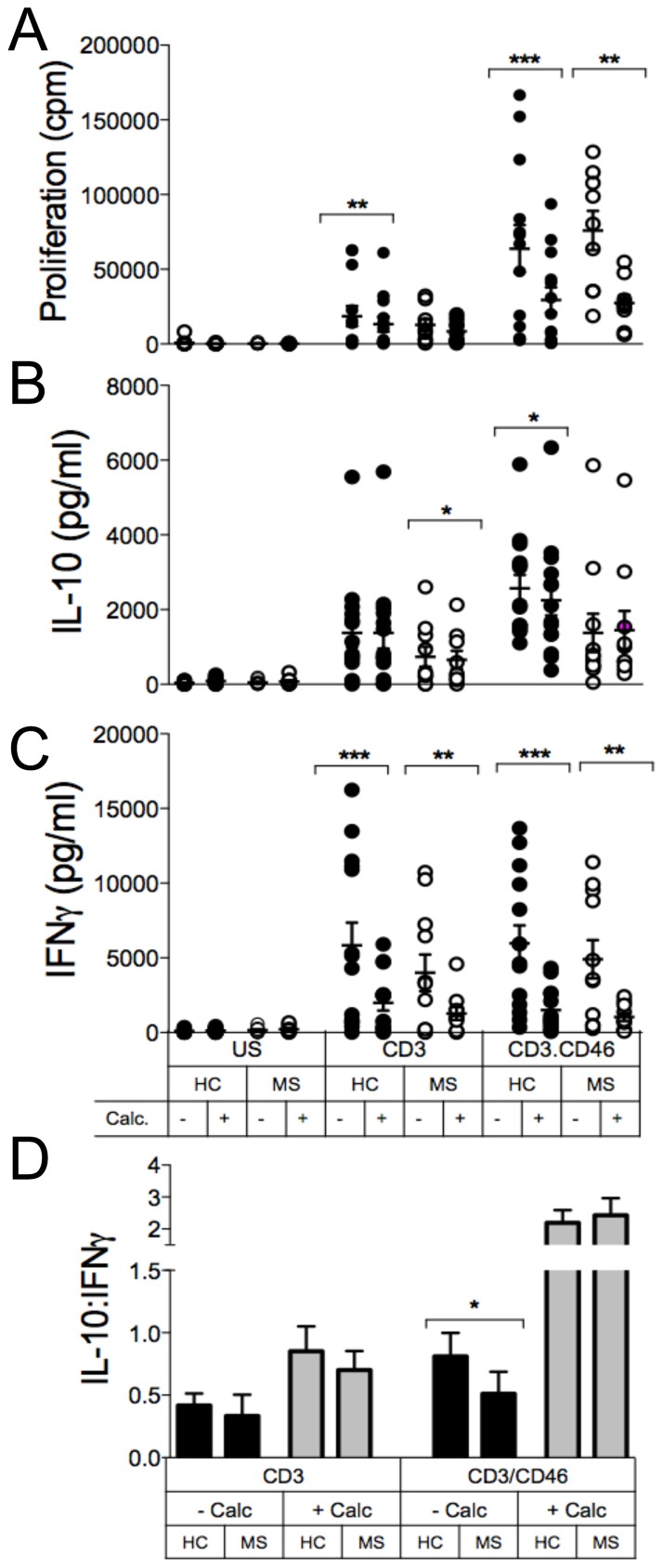
Calcitriol restores the IL-10:IFNγ ratio upon CD46 costimulation of CD4+ T cells in MS. Purified CD4+ T cells from healthy controls (HC) or RRMS patients (MS) were left unstimulated or were stimulated as indicated by immobilized antibodies for 4 days, in presence or absence of calcitriol (10^−7^M). (**A**) Proliferation was determined by ^3^H incorporation. The levels of IL-10 (**B**) and IFNγ (**C**) in the supernatants were determined by ELISA (mean ± SEM; samples were analyzed using the Wilcoxon test). (**D**) The IL-10:IFNγ ratio is also represented to bypass the change related to differences in proliferation (mean ± SEM; samples were compared using the Mann Whitney U-test).

We next determined whether addition of active Vitamin D could modulate CD46 functions first in CD4+ T cells from healthy donors. CD46 costimulation in presence of IL-2 promotes Tr1 differentiation and allows the cells to switch from producing IFNγ to secreting IL-10 [10], [11]. Hence, we assessed the role of calcitriol on IFNγ and IL-10 production. Purified CD4+ T cells were activated with anti-CD3 or anti-CD3/CD46 antibodies with or without IL-2, as a control, and in the presence or absence of calcitriol. After 2 days, production of IL-10 and IFNγ was examined by performing secretion assays (Figure 4A). As expected, CD3/CD46 costimulation promoted IL-10 secretion compared to CD3 activation alone, which was particularly boosted by addition of IL-2. Addition of calcitriol (10^−7^M) strongly increased IL-10 production per cell while it decreased IFNγ secretion (Figure 4A). These data were correlated to the secretion of IL-10 and IFNγ (Figure 4B) in the supernatants determined by ELISA after 4 days. Although lower levels of cytokine were observed by ELISA (due to the lower proliferation rate – see Figure 5A), calcitriol had a stronger effect on IFNγ than IL-10, hence resulting in an increased IL-10:IFNγ ratio.

### Calcitriol restores a normal IL-10:IFNγ ratio in T cells from patients with MS

A defect in IL-10 production upon CD46 costimulation was first identified in patients with RRMS [12]. Hence, we next investigated the effects of calcitriol (10^−7^M) on CD46 function in a cohort of RRMS patients (n = 10) (Figure 5). Addition of calcitriol led to lower proliferation of CD46-costimulated T cells in both cohorts. As expected, CD46-activation induced Tr1 differentiation in CD4+ T cells from healthy donors, visualized by the increase in the IL-10:IFNγ ratio, which was significantly increased by calcitriol. As previously reported [12], MS T cells secreted less IL-10 but similar amounts of IFNγ after CD46 costimulation compared to T cells from healthy donors (Figure 5B and C). Addition of calcitriol had no effect on IL-10 production (while it decreased IL-10 in T cells from healthy donors) but induced a dramatic reduction in IFNγ secretion, resulting in an increased IL-10:IFNγ ratio, similar to the one observed in healthy donors (Figure 5D). Hence, these data demonstrate the ability of calcitriol to restore a correct IL-10:IFNγ ratio upon CD46 stimulation in CD4+ T cells from MS patients.

### The supernatants of CD46-costimulated T cells in presence of calcitriol decrease proliferation of bystander T cells

**Figure 6:**
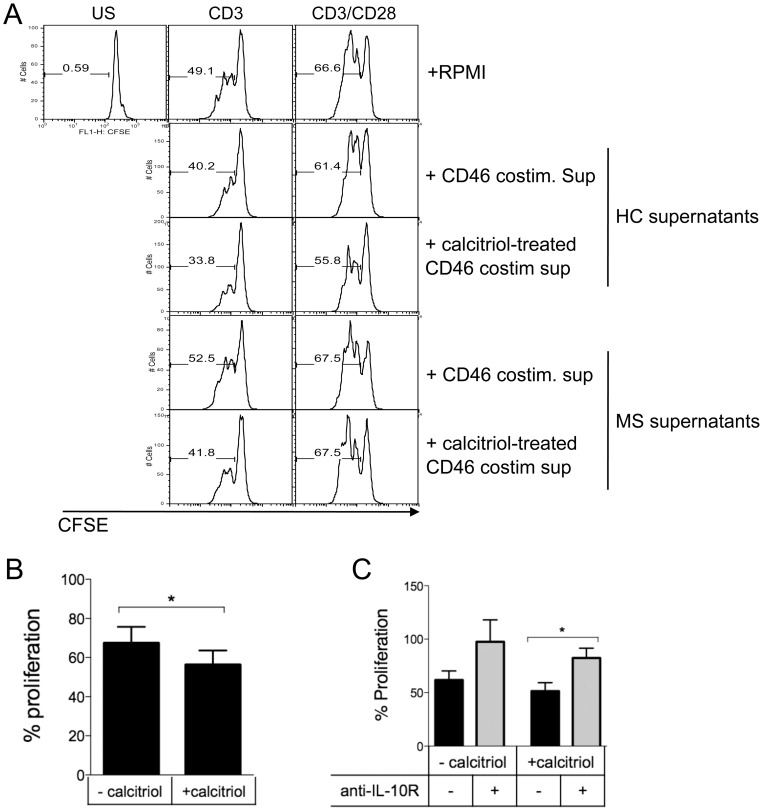
Calcitriol-treated cell supernatants decrease proliferation of bystander CD4+ T cells. (**A**) Purified CD4+ T cells were CFSE pre-labeled and then activated by anti-CD3 or anti-CD3/CD28 antibodies, in presence or absence of culture supernatants from CD46-costimulated T cells with or without calcitriol from either a healthy donor or a patient with MS, as indicated. Proliferation was assessed by flow cytometry after 3 days. (**B**) The data obtained with the supernatants from CD46-costimulated T cells from 3 healthy donors are represented. (**C**) CFSE-labeled naïve T cells were activated by anti-CD3 antibodies (1 µg/ml), in presence of cell supernatants from CD46-costimulated T cells with or without calcitriol (n = 3). A blocking anti-IL-10 antibody or control IgG1 was added to the culture. Proliferation was assessed after 3 days.

As calcitriol promotes secretion of IL-10, an anti-inflammatory cytokine that reduces T cell proliferation [39], we next assessed whether the supernatants from calcitriol-treated cells were able to modulate the activation of bystander T cells. Purified CD4+ T cells were CFSE-labeled and then activated by anti-CD3 or anti-CD3/CD28 antibodies in presence of cell culture supernatants from CD46-costimulated T cells for 5 days in presence or absence of calcitriol from either one healthy donor or one untreated patient with RRMS. After 3 days, proliferation was assessed by flow cytometry (Figure 6A). Proliferation of cells incubated with the supernatants from calcitriol-treated cells had a lower proliferation rate than cells cultured with supernatants from cells activated in absence of calcitriol. Moreover, a decrease in proliferation was also observed when using supernatants from MS T cells (Figure 6A). The reduced proliferation was mostly observed upon CD3 activation, the weaker T cell stimulation. Figure 6B shows the data obtained with the supernatants of CD46-costimulated T cells in presence or absence of calcitriol from 3 healthy donors. Calcitriol has a very short half-life and should have been metabolized after 5 days of culture. Nevertheless, to further demonstrate that IL-10 was at least partly responsible for the lower proliferation observed in presence of calcitriol-treated cell supernatants, we added a blocking anti-IL-10R antibody or control IgG1 in the assay. Naïve T cells were stimulated with anti-CD3 antibodies at 1 µg/ml, in presence of supernatants from CD46-cotimulated T cells. Addition of a blocking anti-IL-10R partly reverted the effects of the supernatants (Figure 6C), suggesting that IL-10 was at least in part responsible for the lower proliferation of the bystander T cells.

### CD46 costimulation and calcitriol modulates CD8+ T cells

**Figure 7:**
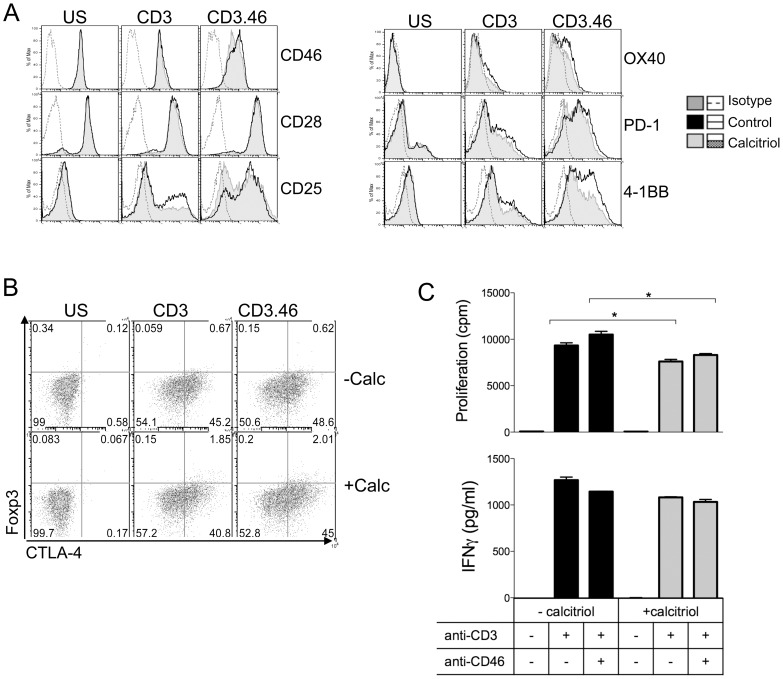
CD46 costimulation and calcitriol modulate CD8+ T cells. (**A**) Purified CD8+ T cells from 3 healthy donors were left unstimulated or stimulated as indicated by immobilized anti-CD3 and anti-CD3/CD46 antibodies, in presence or absence of calcitriol. (**A**) The expression levels of surface CD46, CD28, CD25, OX40, PD-1 and 4-1BB were determined by flow cytometry after 3 days. (**B**) The levels of CTLA-4 and Foxp3 were determined after intracellular staining. (**C**) Proliferation was determined by ^3^H incorporation. The levels of IFNγ in the supernatants were determined by ELISA (mean ± SEM). The data shown for this donor are representative of the three donors.

As the role of CD46 activation on human CD8+ T cells has not been extensively studied, we also examined whether CD46 costimulation affected CD8+ T cells, and whether this was modulated by the addition of calcitriol. Purified human CD8+ T cells (Figure S2) were activated by anti-CD3 or costimulated by anti-CD3/CD46 antibodies in the presence or absence of calcitriol. We first determined whether CD46 costimulation and calcitriol could modulate the phenotype of CD8+ activated T cells. We assessed expression of CD46, CD28, CD25 and Foxp3 but also of other costimulatory molecules such as 4-1BB (CD137), OX40 and of the coinhibitory receptors CTLA-4 and PD-1 to have a broader view of the potential role of CD46 costimulation on CD8+ T cells (Figure 7 and Figure S2). Similarly to what was observed in CD4+ T cells, CD46 costimulation led to its downregulation, which was further increased by addition of calcitriol. CD46 costimulation also led to increased expression of CD28, CD25, OX40, 4-1BB, and PD-1 compared to CD3-activation alone (Figure 7A and Figure S2). In contrast to what was shown in CD4+ T cells, calcitriol had no effect on CD28 and CTLA-4 expression (Figure 7A and 7B, respectively), and decreased CD25, OX40, 4-1BB and PD-1 expression on activated CD8+ T cells. Calcitriol however slightly increased Foxp3 expression but there was no additional effect of CD46 costimulation. Next, we determined proliferation and production of IFNγ (Figure 7C). CD46 costimulation did not profoundly affect CD8+ T cell responses in this *in vitro* model, although CD46 costimulation slightly promoted CD8+ T cell proliferation compared to CD3 activation, and calcitriol decreased the proliferation of both CD3- and CD3/CD46 activated T cells. Similarly, CD46 costimulation only slightly decreased IFNγ production (Figure 7A), and we were unable to detect any IL-10 in the supernatants of activated CD8+ T cells (not shown). Calcitriol had no significant effect on IFNγ production. Therefore, CD46 costimulation modulates the phenotype of CD8+ T cells and calcitriol decreases expression of most of the costimulatory molecules tested for activated CD8+ T cells.

## Discussion

T cell activation is essential to ensure a proper immune response, and costimulatory molecules are required to exert this effect. While CD28 has been identified decades ago as a major costimulatory molecule for T cells, the identification of a role of CD46 in T cell costimulation is more recent [4] but it is now well established that CD46 contributes on its own to regulate T cell activation and differentiation. CD46 can notably promote Tr1 differentiation, and switch T cells from a Th1 to a Tr1 phenotype depending on the IL-2 present in the milieu, acting therefore as a sensor for inflammation [10], [11]. Moreover, CD46 cell surface expression on T cells is tightly controlled [9], [40]. Its ectodomain is shed upon CD46 triggering, followed by the processing of its two cytoplasmic tails. This provides an on/off signal to first activate, and then deactivate T cells [9]. Hence, CD46 plasticity is crucial to ensure proper T cell homeostasis, and regulation of its expression provides a means to control its functions. Importantly, activated T cells produce some C3b, a ligand for CD46, that may therefore act as a ligand for autocrine stimulation *in vivo* [11]. Importantly, the CD46 pathway is altered in an increasing number of human pathologies. A defect in IL-10 production was first identified in patients with MS [12], [13], [14], followed by a report of defective IL-10 production in patients with asthma [15] and altered cytokine production was shown in patients with rheumatoid arthritis [11]. The fact that CD46 is altered in several pathologies highlights its importance in controlling T cell functions. It also underlines the fact that we need to better understand the molecular mechanisms that regulate its expression.

Vitamin D deficiency has long been correlated with the high incidence of several human diseases in countries with Northern latitudes [41]. This notably includes MS, asthma and RA, pathologies in which the CD46 pathway is defective. Therefore, Vitamin D supplementation is considered as a potential immunomodulatory therapy. Here, we report that the active metabolite of Vitamin D affects CD46-mediated T cell activation by controlling CD46 expression and function of both CD4+ and CD8+ T cells.

We show that, after addition of calcitriol, CD46 expression levels are initially slightly increased in unstimulated CD4+ T cells and CD3-activated but decreased upon longer activation period when CD46 is ligated. The increased CD46 expression might lower the threshold for CD46 activation of cells, resulting in a higher incidence of IL-10-secreting cells. CD46 ectodomain is shed by a matrix-metallo-protease (MMP)-dependent cleavage upon CD46 triggering on T cells [8], [9]. It has been shown that calcitriol upregulates MMP-3 expression in chondrocytes [42], but downregulates MMP levels in tuberculosis-infected leukocytes [43]. Hence, modulation of CD46 by calcitriol could be the result of an indirect effect on MMP levels [9], [44], [45]. Strikingly, the decrease in CD46 expression observed upon CD46 costimulation was correlated with a strong increase in CD28 levels. This effect was however CD46 dependent, as no effect of calcitriol on CD28 expression was noticed when CD4+ T cells were costimulated by CD3/CD28 (not shown). It has been reported that CD86, one of the ligands of CD28, is a potential target of calcitriol [46]. Our data suggest that, by increasing specifically CD28 expression on CD46-activated T cells, calcitriol participates in a crosstalk between these two costimulatory molecules, rendering these cells responsive to further interaction with CD86-expressing APCs. Moreover, as calcitriol mostly decreases proliferation of CD46-activated T cells but has a lesser effect on CD3/CD28/CD46 costimulated T cells (data not shown), the increase in CD28 expression might also be a means to prevent lower cell proliferation in the presence of calcitriol. We also show that calcitriol enhances CD25, Foxp3 and CTLA-4 expression on CD46 costimulated CD4+ T cells isolated from healthy donors. The increase in CD25 might correlate with the ability of calcitriol to switch T cells from a Th1 to a Tr1 phenotype in presence of IL-2, rendering them responsive to IL-2 by upregulation of its receptor. Importantly, we observed similar trends when calcitriol was added to purified naïve T cells (Figure S3). Strikingly, in contrast to what was observed in T cells from healthy donors, and although CD46 costimulation induced CD25 expression in MS T cells, addition of calcitriol led to a strong decrease in CD25 expression in these cells. Levels of Foxp3 were lower than in healthy T cells. CTLA-4 expression was not affected, and calcitriol decreased CD46 at the surface of MS T cells in similar fashion than in healthy T cells. Importantly, when the same MS T cells were co-activated by CD3/CD28, CD25 expression was either increased or unaffected but not decreased, suggesting a specific effect on the CD46 pathway (Figure S1). The regulation of CD28 was normal in most MS T cells but we observed a decrease in CD28 expression for 4 out of 11 patients. Importantly, in this pilot study, a similar trend was observed for both IFN beta-treated and untreated patients although future studies should be performed with larger cohorts. It is worth noting that polymorphisms in CD25 [47] and in proteins involved in the vitamin D pathway [48] have been identified in MS, which might at least partly be relevant to the altered regulation of CD25 expression in MS. It is also possible that this mechanism has relevance to the action of vitamin D in MS, as vitamin D supplementation has been linked to a possible reduction in disease activity as measured by MRI correlates [49] and early clinical trials have shown that anti-CD25 immunotherapy shows promise in reducing the number of MS relapses [50]. Further investigations should therefore be performed to fully understand the molecular mechanisms involved in the regulation of CD25 expression by calcitriol in CD46-costimulated T cells.

We also found that calcitriol decreased T cell proliferation of CD46-costimulated CD4+ T cells as previously reported for CD28 costimulation [51], and so in both healthy donors or RRMS patients. It is known that calcitriol inhibits proliferation by inhibiting IL-2 production [21] as the *IL-2* gene contains a negative calcitriol response element (VDRE) in its promoter [52]. However CD46 activation requires exogenous IL-2, that antagonizes the effect of calcitriol on proliferation [51]. Moreover, a recent study has shown that calcitriol affected more the proliferation of CD4+CD25+Foxp3+ nTreg than conventional responders T cells [53]. Interestingly, the authors also observed that calcitriol induced a population of IL-10 secreting T cells. No difference in proliferation was observed between T cells from healthy donors or from patients with MS.

In order to provide a broader picture of the effect of calcitriol in the CD46 pathway in T cells, we also studied CD8+ T cells. Indeed, CD8+ T cells have been reported to express more CD46 than CD4+ T cells [54]. CD46 costimulation of CD8+ T cells led to increased proliferation and induced expression of several markers involved in the regulation of T cell activation (CD28, CD25, OX40, PD-1). Proliferation of CD8+ T cells was modulated by calcitriol as previously reported [55], although calcitriol affected CD8+ less than CD4+ T cells in our *in vitro* model. Strikingly, calcitriol had different effects on the phenotype of CD4+ and CD8+ T cells, as in CD8+ T cells, it decreased CD25 and had no effect on either CD28 or CTLA-4 expression. Moreover, addition of calcitriol led to a decreased expression of most of the costimulatory molecules tested, suggesting that it lowers CD8+ activation overall. Indeed, CD46 ligation was shown to reduce CD8+ cytotoxic function in a CD46 transgenic mouse model [5]. Further studies will be necessary to fully elucidate the role of CD46 on human CD8+ T cell functions.

As already reported, CD46-activation of CD4+ T cells induces Tr1 differentiation, demonstrated by the increase in the IL-10:IFNγ ratio, which was significantly enhanced by calcitriol. It was shown that, in presence of IL-2, CD46-activated T cells lower their IFNγ production in favor of IL-10 secretion, switching from a Th1 toward a Tr1 phenotype [11]. Our data indicate that calcitriol promotes this switch by not only reducing IFNγ production but also promoting IL-10 secretion upon CD46 costimulation. While, as previously reported, we observed a decreased IL-10 production in T cells from MS patients, addition of calcitriol could restore the overall IL-10:IFNγ ratio, by maintaining the IL-10 levels (which are lowered in healthy controls and correlated with the reduced proliferation observed with calcitriol) and by promoting a further decrease in IFNγ production. These data suggest that the decrease in CD25 expression observed in MS T cells upon addition of calcitriol does not impede the beneficial effect of calcitriol on cytokine production, at least *in vitro*. Calcitriol has previously been shown to correct defective IL-10 production in steroid resistant asthma patients [30], [31], and *in vitro* studies have shown an effect on T cells isolated from patients with Crohn's disease [56]. Our data now demonstrate the ability of calcitriol to affect the CD46 pathway and suggest that it can restore a correct IL-10:IFNγ ratio upon CD46 costimulation in CD4+ T cells from MS patients. Moreover, the supernatants of CD46-costimulated T cells in presence of calcitriol further increased their ability to suppress the proliferation of bystander T cells. Together, these data further support the role of calcitriol in promoting a regulatory phenotype upon CD46-activation, expressing higher levels of regulatory receptors and mainly secreting IL-10. As Vitamin D appears to be an important factor for several human pathologies, it will now be crucial to further investigate this pathway not only in MS but also in patients with other chronic inflammatory diseases.

## Supporting Information

Figure S1 **Calcitriol promotes CD25 expression upon CD46 costimulation of CD4+ T cells but not in those from patients with MS.** Purified CD4+ T cells from healthy controls or RRMS patients were left unstimulated or stimulated by immobilized anti-CD3, anti-CD3/CD28 or anti-CD3/CD46 antibodies in presence of calcitriol (10^−7^M) or ethanol, for 5 days. Expression of CD25 was then monitored by flow cytometry. Representative data showing CD25 expression for one healthy donor (HC) or 2 MS donors (MS#1 and MS#2) are shown.
(TIF)

Figure S2 **CD46 costimulation and calcitriol modulate the phenotype of CD8+ T cells.** CD8+ T cells from 3 healthy donors (purification shown in (**A**)) were activated by anti-CD3 or anti-CD3/CD46 antibodies, in presence or absence of calcitriol. (**B**) The levels of CD46, CD28, CD25, OX40, 4-1BB, PD-1, CTLA-4 and Foxp3 was assessed by flow cytometry after 3 days. The normalized MFIs (to control samples) are shown for the 3 donors, and the samples analyzed using the paired Student's t-test.
(TIF)

Figure S3 **Calcitriol induces similar phenotypic changes in naïve T cells and CD4+ T cells.** Naïve CD4+ T cells (representative purification shown in (**A**)) were activated by anti-CD3 or anti-CD3/CD46 antibodies, in presence or absence of calcitriol for 4 days. (**B**) The levels of CD28, CD25, CTLA-4 and Foxp3 were then analyzed by flow cytometry and the average MFI obtained for 3 donors is represented.
(TIF)
