# Supplementary figures and images for: Calcitriol Modulates the CD46 Pathway in T Cells
Source: PLoS One. 2012 Oct 29;7(10):e48486. doi: 10.1371/journal.pone.0048486 (PMC3483209; doi:10.1371/journal.pone.0048486)

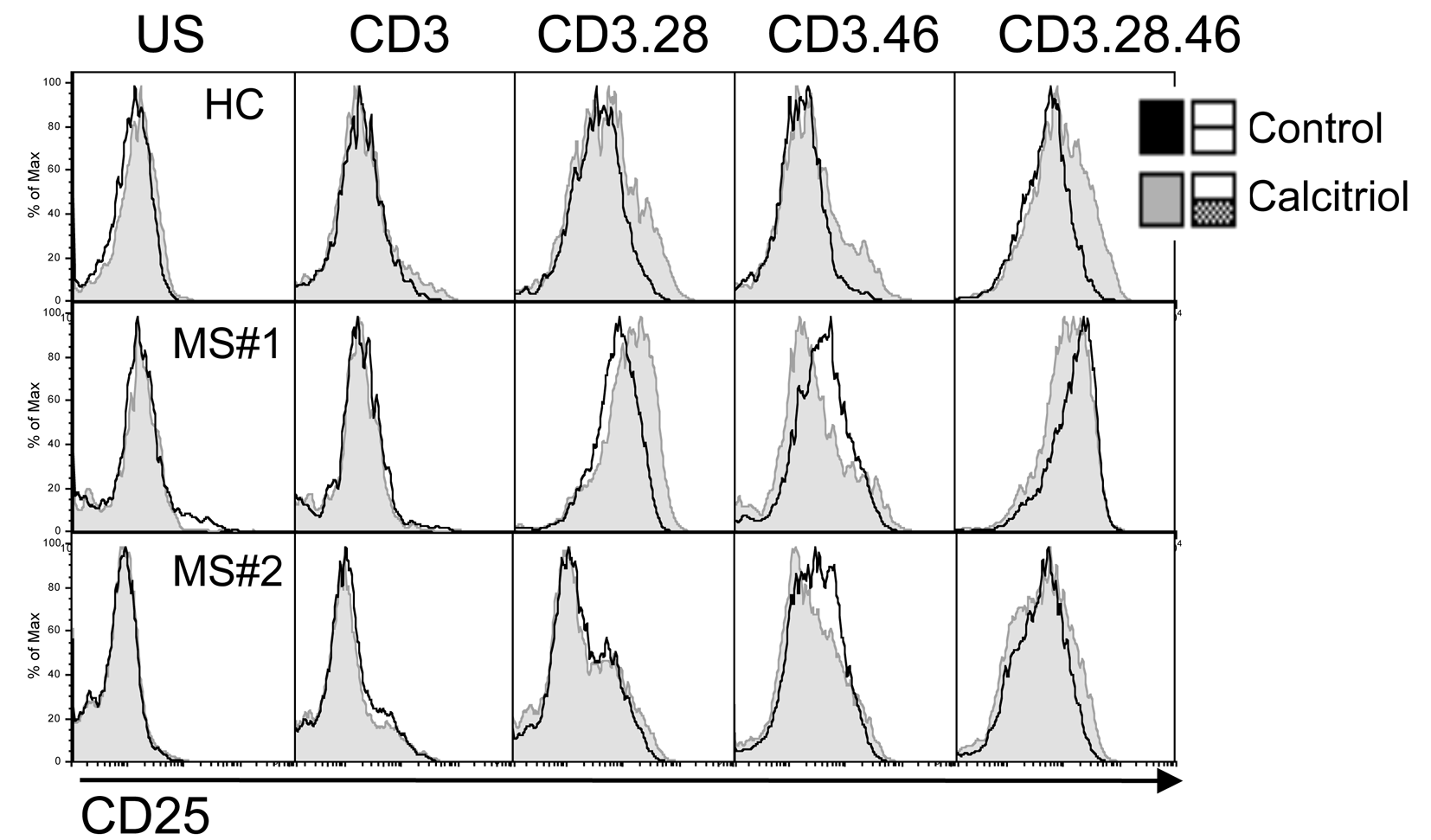

Supplement: Figure S1 — Calcitriol promotes CD25 expression upon CD46 costimulation of CD4+ T cells but not in those from patients with MS. Purified CD4+ T cells from healthy controls or RRMS patients were left unstimulated or stimulated by immobilized anti-CD3, anti-CD3/CD28 or anti-CD3/CD46 antibodies in presence of calcitriol (10−7M) or ethanol, for 5 days. Expression of CD25 was then monitored by flow cytometry. Representative data showing CD25 expression for one healthy donor (HC) or 2 MS donors (MS#1 and MS#2) are shown. (TIF) [file pone.0048486.s001.tif]

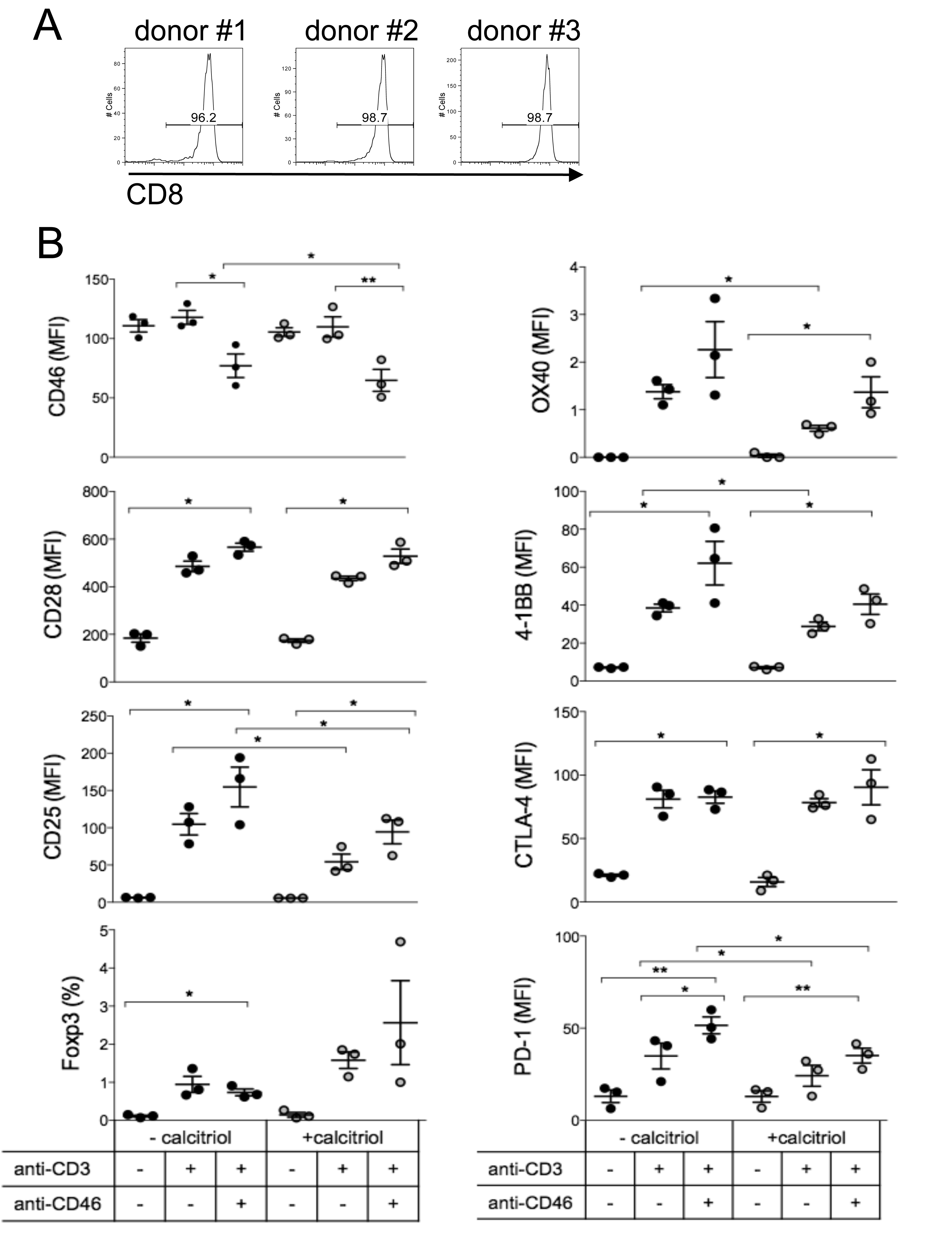

Supplement: Figure S2 — CD46 costimulation and calcitriol modulate the phenotype of CD8+ T cells. CD8+ T cells from 3 healthy donors (purification shown in (A)) were activated by anti-CD3 or anti-CD3/CD46 antibodies, in presence or absence of calcitriol. (B) The levels of CD46, CD28, CD25, OX40, 4-1BB, PD-1, CTLA-4 and Foxp3 was assessed by flow cytometry after 3 days. The normalized MFIs (to control samples) are shown for the 3 donors, and the samples analyzed using the paired Student's t-test. (TIF) [file pone.0048486.s002.tif]

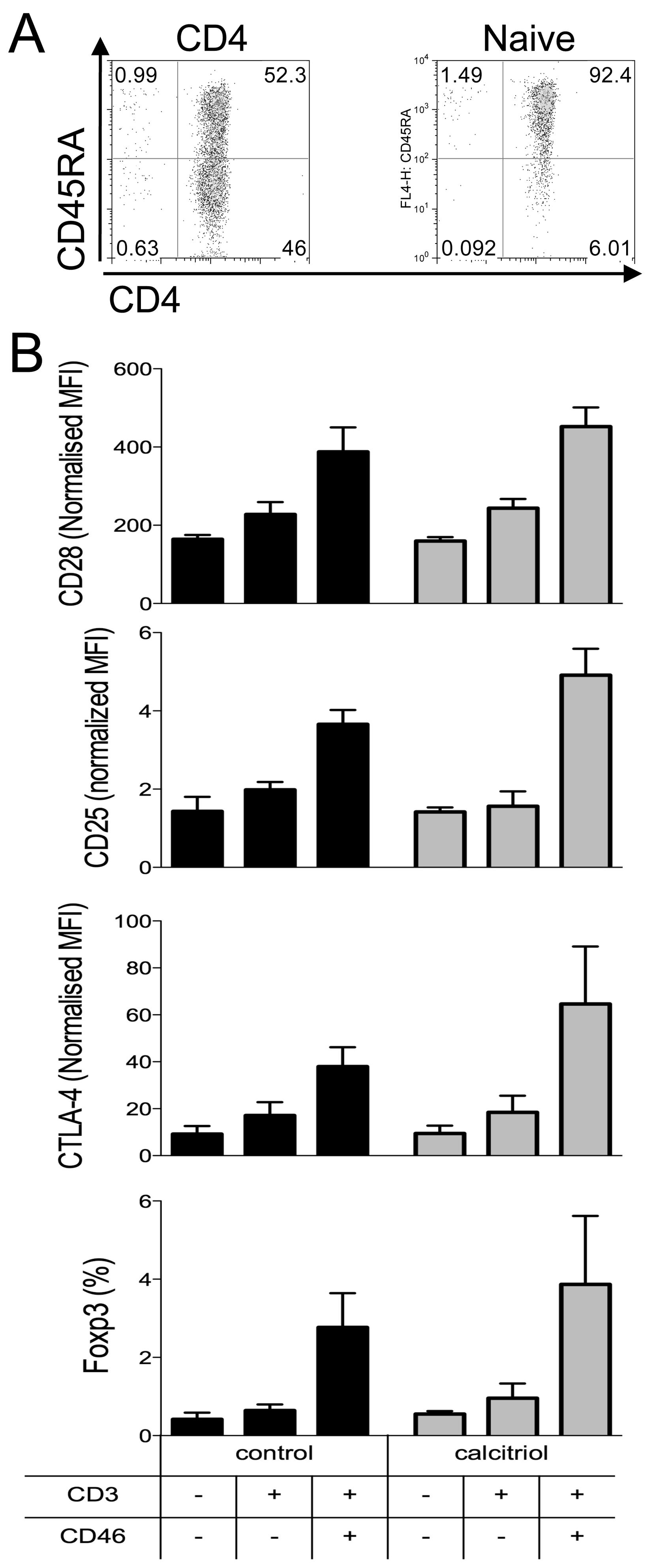

Supplement: Figure S3 — Calcitriol induces similar phenotypic changes in naïve T cells and CD4+ T cells. Naïve CD4+ T cells (representative purification shown in (A)) were activated by anti-CD3 or anti-CD3/CD46 antibodies, in presence or absence of calcitriol for 4 days. (B) The levels of CD28, CD25, CTLA-4 and Foxp3 were then analyzed by flow cytometry and the average MFI obtained for 3 donors is represented. (TIF) [file pone.0048486.s003.tif]
